# Method for Classifying Schizophrenia Patients Based on Machine Learning

**DOI:** 10.3390/jcm12134375

**Published:** 2023-06-29

**Authors:** Carmen Soria, Yoel Arroyo, Ana María Torres, Miguel Ángel Redondo, Christoph Basar, Jorge Mateo

**Affiliations:** 1 Institute of Technology, University of Castilla-La Mancha, 16071 Cuenca, Spain; 2 Clinical Neurophysiology Service, Virgen de la Luz Hospital, 16002 Cuenca, Spain; 3 Faculty of Social Sciences and Information Technology, University of Castilla-La Mancha, 45600 Talavera de la Reina, Spain; 4 School of Informatics, University of Castilla-La Mancha, 13071 Ciudad Real, Spain; 5 Faculty of Human and Health Sciences, University of Bremen, 28359 Bremen, Germany

**Keywords:** shizophrenia, mental disorders, machine learning, artificial intelligence, biomedical signals

## Abstract

Schizophrenia is a chronic and severe mental disorder that affects individuals in various ways, particularly in their ability to perceive, process, and respond to stimuli. This condition has a significant impact on a considerable number of individuals. Consequently, the study, analysis, and characterization of this pathology are of paramount importance. Electroencephalography (EEG) is frequently utilized in the diagnostic assessment of various brain disorders due to its non-intrusiveness, excellent resolution and ease of placement. However, the manual analysis of electroencephalogram (EEG) recordings can be a complex and time-consuming task for healthcare professionals. Therefore, the automated analysis of EEG recordings can help alleviate the burden on doctors and provide valuable insights to support clinical diagnosis. Many studies are working along these lines. In this research paper, the authors propose a machine learning (ML) method based on the eXtreme Gradient Boosting (XGB) algorithm for analyzing EEG signals. The study compares the performance of the proposed XGB-based approach with four other supervised ML systems. According to the results, the proposed XGB-based method demonstrates superior performance, with an AUC value of 0.94 and an accuracy value of 0.94, surpassing the other compared methods. The implemented system exhibits high accuracy and robustness in accurately classifying schizophrenia patients based on EEG recordings. This method holds the potential to be implemented as a valuable complementary tool for clinical use in hospitals, supporting clinicians in their clinical diagnosis of schizophrenia.

## 1. Introduction

The psychiatric disorder schizophrenia is recognized to have a substantial impact on life expectancy, reducing it by approximately 15 to 20 years compared to the general population. Several factors contribute to the decreased life expectancy in individuals with schizophrenia. Unhealthy lifestyle choices, such as poor diet, lack of physical activity, and higher rates of smoking, along with a significantly elevated risk of suicide (about 12 times higher), collectively contribute to this overall reduction in lifespan. In turn, schizophrenia is a lifelong condition, persisting at a prevalence rate ranging between 0.3% and 0.66% in the population [1].

Schizophrenia impacts approximately 1% of the global population at some stage in their lives. This corresponds to an estimated global population of around 20 million individuals living with schizophrenia. Additionally, the average worldwide incidence rate of schizophrenia is estimated to be approximately 15 cases per 100,000 people per year, being considered one of the leading causes of disability globally. The disability attributed to schizophrenia contributes to approximately 1% of the global burden of disease.

It should be noted that schizophrenia can exhibit a variable course, characterized by episodes of symptom exacerbation (acute episodes) and periods of partial or complete remission. The severity of symptoms and the functional impact on daily life can vary significantly among individuals affected by schizophrenia. Globally, individuals with schizophrenia encounter difficulties in accessing sufficient mental health services, including timely diagnosis, treatment, and ongoing support. Factors such as limited resources, insufficient number of trained mental health professionals, and social stigma can impede access to essential care for individuals with schizophrenia [2,3].

Schizophrenia research has made significant advancements in recent years, and the utilization of the electroencephalogram (EEG) in studying schizophrenia has played a crucial role in this progress. The EEG is a non-invasive technique that measures the electrical activity of the brain, providing valuable information about various behavioral, pathological, and drug-related patterns. As a result, the medical application of EEG has significantly expanded and is now recognized as a crucial component in the study of brain behavior, as well as the assessment and treatment of brain-related diseases. The study and diagnosis of mental pathologies are of utmost importance in investigating the behavioral characteristics of the brain in affected patients.

The application of automatic analysis techniques for EEG recordings can serve as an additional tool to complement visual EEG analysis [4,5,6]. The study of EEG recordings has proven valuable for psychiatric departments, as these techniques have been instrumental in estimating the incidence, facilitating treatment decisions, and aiding in the diagnosis of various brain pathologies. Furthermore, the utilization of classification tools in research plays a crucial role in improving medical problem amelioration and diagnostic aids. A wide range of techniques has been implemented for classification purposes, including neural networks, expert systems, linear programming, evolutionary algorithms, machine learning (ML), deep learning, and swarm intelligence [7,8,9,10,11,12,13]. Many existing works in the literature apply these techniques with different purposes and with great success. For example, ref. [14] focuses on the detection of driving fatigue using a multifractal theory-based approach, where the authors employ sensor data from various sources to develop a feature detection method; Ref. [15] concentrates on visual tracking using a Siamese-oriented region proposal network, addressing the problem of tracking objects accurately and efficiently in videos; Ref. [16] investigates the therapeutic potential of transcranial alternating current stimulation (tACS) for depression treatment, with the aim to assess the efficacy of tACS; whilst [17] introduces a deep learning-based predictive evaluation system for smart healthcare applications, leveraging deep learning techniques to enhance the predictive capabilities of healthcare systems. However, while these articles contribute to various domains and highlight advancements in their respective fields, they do not directly compare to the specific context of EEG analysis for schizophrenia classification based on ML methods.

The widespread adoption of automatic EEG analysis has been driven by several factors, including the robustness of ML algorithms, technological advances, the increasing availability of data, and the affordability of high-performance computing [18]. In the literature, various classification methods are commonly employed, including neural networks [18,19,20,21,22,23] and Support Vector Machines (SVM) [24,25,26,27,28]. Other methods found to be valid for data classification are K-Nearest Neighbour (KNN) [18,24,29,30,31], Gaussian Naïve Bayes (GNB) [18,32,33], Adaptive boosting (Adaboost) [18,24,34,35] and Decision Tree (DT) [18,24,36].

This work focuses on the classification of patients with schizophrenia using an eXtreme Gradient Boosting (XGB) algorithm. XGB, a supervised algorithm and a variant of gradient boosting, is employed for its ability to enhance both speed and execution in the ML model. The development of the XGB algorithm has revolutionized the field by introducing a range of essential properties. Firstly, its system scalability allows for seamless integration with large-scale datasets, making it an ideal choice for handling big data analytics. Secondly, XGB possesses remarkable parallel computing capability, enabling it to leverage multi-core processors and distributed computing frameworks to expedite complex computations. Moreover, the algorithm incorporates regularization techniques, enhancing its ability to prevent overfitting and improve generalization on diverse datasets. Additionally, XGB is renowned for its high-performance optimization, ensuring efficient resource utilization and swift execution, even when dealing with extensive feature spaces. Collectively, these properties position XGB as a versatile and powerful algorithm that significantly advances the capabilities of ML systems [37,38,39]. This system was designed to implement an algorithm that aids clinicians in diagnosing schizophrenia based on EEG recordings. The results obtained from the proposed method have demonstrated its superior performance, surpassing other ML methods in accurately classifying schizophrenia patients from EEG recordings. Thus, this system offers promising potential to enhance the diagnostic process and improve the accuracy of schizophrenia diagnosis in clinical settings.

The document is structured into distinct sections. Section 2 introduces the materials utilized in the study. The Section 3 provides a detailed description of the proposed method. Section 4 presents the obtained results. Section 5 and Section 6 delve into the discussion and conclusion, respectively, offering an analysis of the findings and summarizing the overall study outcomes.

## 2. Material

To conduct this study and assess the performance of the ML system accurately, real EEG recordings were employed. A total of 310 patients, all residents of the province of Cuenca in Spain, were included for this purpose. Following the provision of detailed information regarding the required procedures and obtaining written consent from the participants, the study received approval from the Clinical Research Ethics Committee of the Cuenca Health Area. A total of 205 individuals were included in the study as healthy participants undergoing diagnostic EEG testing for brain disorders. The remaining participants were euthymic patients diagnosed with schizophrenia, and they were recruited from the Serious Mental Disorders Programme of the Psychiatry Service at Hospital Virgen de la Luz in Cuenca. EEG recordings were captured at the Psychiatry Department of the hospital using the Brain Vision 32-channel system, with a sampling frequency of 500 Hz. The placement of electrodes followed the 10–20 International System, conducted by the medical staff responsible for the study.

Table 1 shows the clinical and demographic characteristics of the subjects participating in the study.

**Figure 1:**
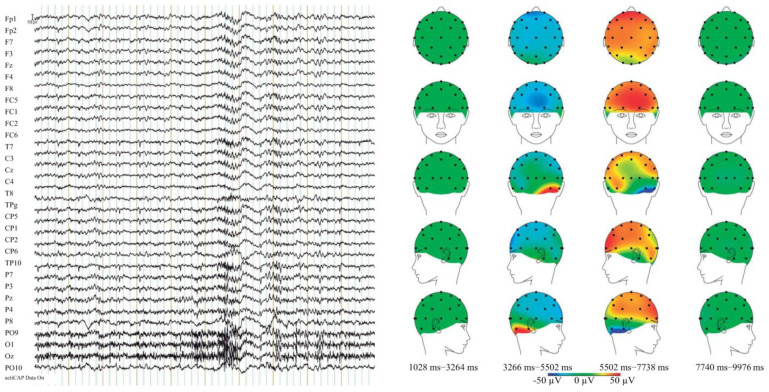
The figure shows the recording of an electroencephalogram and scalp maps. The colors in the scalp map indicate the intensity of the electrical activity recorded at each electrode location (Blue: Low electrical activity or baseline; Green: Mild activity; Yellow: Moderate activity; Orange: High activity; Red: Very high activity).

**Figure 2:**
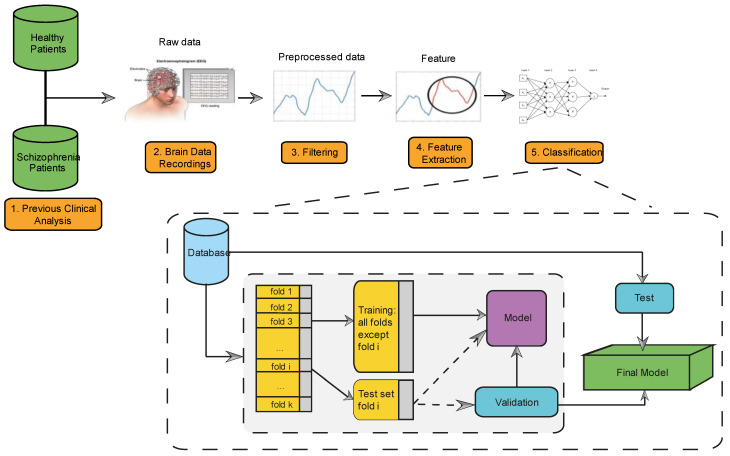
The figure shows the processes followed in this study for the classification of patients with schizophrenia.

After obtaining the EEG recordings from various patients, it was observed that they exhibited instances of muscle noise, artifacts, and baseline fluctuations. To enhance the accuracy of the results, a filtering process was performed using the proposed XGB method [6,40]. This filtering step aimed to refine the EEG data by reducing unwanted noise and artifacts, thus improving the overall quality of the results obtained through the XGB classification algorithm. An example of an EEG recording, accompanied by scalp maps, is depicted in Figure 1. The creation of these maps is facilitated by information regarding the electrode positions. The maps display the distribution of voltage on the head in either the time or frequency domain, with different colors representing varying voltage levels. In this study, the algorithm employed for map creation is based on the interpolation of spherical splines, as described in [41]. Parameters such as the spline order and the maximum degree of the Legendre polynomial are utilized to calculate and generate the maps. Figure 2 provides a visual representation of the proposed methodology, highlighting three essential steps that follow the initial clinical analysis (Figure 2 (1)) and brain data recordings (Figure 2 (2)). The first step involves pre-processing the EEG recordings to eliminate interference and noise (Figure 2 (3)). The second step entails calculating relevant features for each EEG channel (Figure 2 (4)) Finally, in the last step, after constructing the study database, schizophrenia is classified using ML methods (Figure 2 (5)).

## 3. Method

XGB is a predictive algorithm that employs boosting and operates under supervised conditions. Boosting involves sequentially generating multiple “weak” prediction models, which utilize the results of the previous model to create a model with “stronger” predictive power and result stability. “Stronger” in the context of boosting refers to the improved predictive power and stability of the model compared to the individual weak models. When boosting generates a model that is stronger, it means that the final model produced by the boosting algorithm has a higher overall performance and accuracy in predicting outcomes. To achieve a more robust model using these weaker models, an optimization algorithm called Gradient Descent is employed. During the training process, the parameters of each weak model are iteratively adjusted in an attempt to minimize an objective function [42,43]. Because of these traits, the XGB method was selected for constructing the classification scheme for individuals with schizophrenia. When provided with a dataset *S* = *x_j_*, *y_j_*, the XGB method was designed as

(1) $$\hat{y_{j}}=\sum_{p=1}^{P}t_{p}\left(x_{j}\right)$$

where *x_j_* is the input with *m* time variables, *y_j_* represents the output, $\hat{y_{j}}$ shows the predicted output, *t_p_* represents a tree with leaf weight *w_p_* and structure *u_p_*, *j* = 1; 2; …; *n*, and *P* corresponds to the amount of trees.

The regularized objective function for the suggested method is presented in Equation (2). In this instance, it differs from that of Ensemble methods. The suggested method employs a second-order Taylor expansion to estimate the target function of XGB, thus enhancing the prediction accuracy [42,43].

(2) $$R=\sum_{j}r\left(\hat{y_{j}},y_{j}\right)+\sum_{p}\Psi \left(t_{p}\right)$$

(3) $$\Psi \left(t_{p}\right)=\lambda f_{p}+\frac{1}{2}\gamma {\left\|\omega_{p}\right\|}^{2}$$

where *R*() is a function that determines the difference between the target output *y_j_* and the expected output $\hat{y_{j}}$, and which penalizes the complexity of the method. The function *Ψ*() punishes the complexity of the method. To regulate the complexity of the method and avoid overfitting, a regulation term, represented by the weights, is used as a monitoring mechanism. As depicted in Equation (3), *F* corresponds to the tree trimming used to control overfitting, and illustrates the number of leaves in the tree; *λ* represents the learning rate; whilst *w* corresponds to the vector of scores assigned to the leaves. The parameter *γ* is employed to regulate the weight of the system’s complexity [42,43]. In an effort to enhance performance, this study aims to minimize Equation (2).

In turn, this study evaluated the proposed method using various ML techniques for classifying patients into two categories: those with schizophrenia and those without. The techniques compared include DT [24,36], GNB [32,33], KNN [24,29,31], SVM [24,25,44,45] and the suggested method [38,39]. The evaluation was performed using the MatLab statistical and machine learning toolbox (Matlab 2021a). Five-fold cross-validation was applied to prevent overfitting. The database was divided into two groups, with 70% used for training and 30% for testing, and patients were not shared between groups. The phases of the complete study are outlined in Figure 2 (5). It began by selecting the patients to be included, followed by the creation of the database. Then, the training phase was initiated and, finally, the ML models were validated.

Two performance measures were utilized to maximize the results: the accuracy and the area under the curve. Furthermore, to account for the stochastic nature of ML, 100 iterations were carried out randomly to reduce the impact of noise in the data, calculate suitable values and obtain statistically significant results, as stated in [24].

### 3.1. Feature Extraction

The following techniques were employed to extract features from EEG recordings:

- Detrended Fluctuation Analysis (DFA) provides information about the temporal correlations in EEG recordings [46].
- Approximate Entropy (ApEn), for measuring the complexity of the system. The ApEn value is indicated by a positive number, which represents the complexity of the EEG signal [47].
- Hurst exponent, which is used to analyze the behavior of a system over time and determine the existence of fractal series [48].
- Higuchi analyzes the fractal dimension of EEG signals and measures their self-similarity and complexity [49].
- Lyapunov Exponent (LE), which characterizes the rate of separation of close trajectories of a system [50].
- EEG band power, obtained by applying Butterworth filters and calculating the power spectrum of each frequency band (delta (0.5–4 Hz), theta (4–8 Hz), alpha (8–13 Hz) and beta (13–30 Hz)), using the Welch method [51].

### 3.2. Performance Evaluation

In this study, the following evaluation metrics were utilized to measure performance: Specificity (SP), Precision, Degenerate Younden Index (DYI), Recall (also known as Sensitivity), Balanced accuracy, Receiver Operating Characteristic (ROC) and AUC [52,53,54].

## 4. Results

In this section, the discussion will revolve around the results obtained from the EEG recordings utilized for training and validation purposes. The main objective was to evaluate and compare the performance of the XGB system with other frequently employed classification systems documented in the literature.

**Figure 3:**
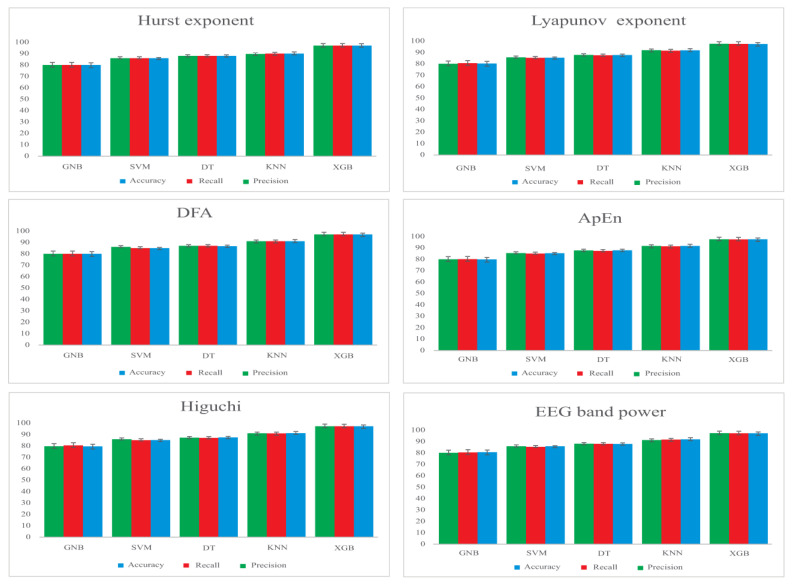
The figure presents the results attained with different machine learning algorithms for various features.

The training results of ML techniques using different features individually and in combination are depicted in Figure 3. The figure clearly illustrates that the proposed system outperforms other methods, showcasing superior classification accuracy and effectiveness.

Table 2 displays a comparison of various classification methods, including SVM, DT, GNB, KNN, and the proposed system. The performance metrics for classifying schizophrenia patients and healthy individuals are presented in the table, allowing for a comprehensive assessment of the different methods’ effectiveness. Comparing the performance of different classification models, it is evident that SVM and GNB exhibit lower classification values with an accuracy of around 86%. DT and KNN show improvement, achieving accuracy values of 89%. However, the proposed XGB method outperforms all other models, attaining significantly higher accuracy close to 94%. Although KNN and DT exhibit similar Precision and Recall values to the proposed method, they do not achieve the same level of accuracy. SVM and GNB achieve F1 score values close to 87%, but they fall short of the performance levels achieved by the proposed method.

The performance of the proposed XGB model was evaluated in the classification of the two analyzed classes, using commonly used parameters in the literature such as Kappa, MCC, AUC, and DYI index. The Matthews Correlation Coefficient (MCC), known as the most reliable statistical index, was specifically employed for this purpose. The MCC coefficient produces a high score only when the predictions perform well across all four categories of the confusion matrix (TP, FN, TN, and FP), considering the proportions of positive and negative items in the dataset. The obtained MCC values, as shown in Table 3, indicate that the suggested XGB method outperformed the other analyzed methods. While DT and KNN performed relatively well compared to other methods, their performance fell short of the XGB model. A similar trend is observed for the Kappa index, where XGB demonstrated the highest performance among the compared methods, surpassing both KNN and DT.

**Figure 4:**
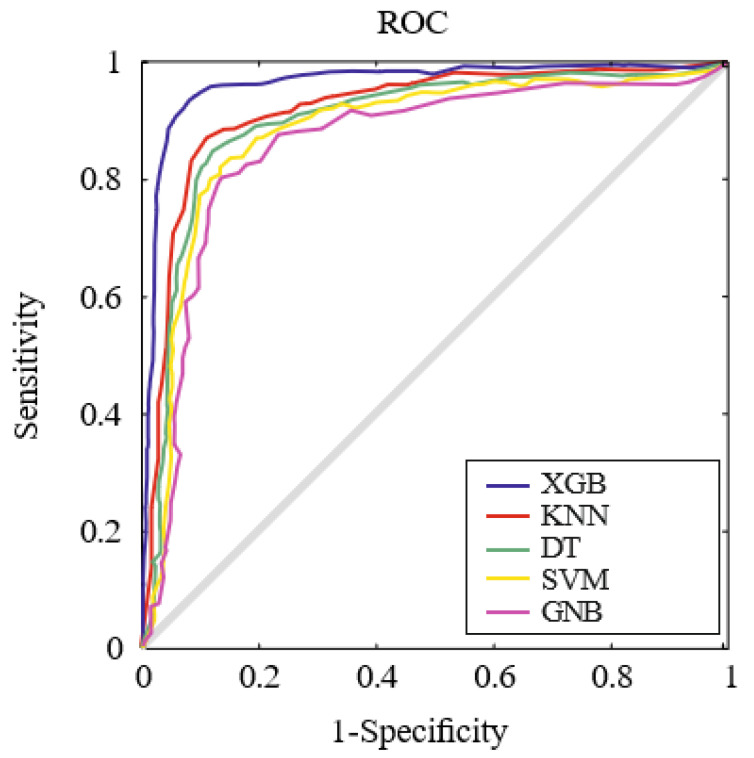
The figure depicts the different ROC curves for the five machine learning algorithms evaluated.

The receiver operating characteristic (ROC) analysis was utilized to compare the classification capability of the proposed method. The ROC curve is generated by plotting the sensitivity against the specificity for various threshold values. Both Figure 4 and Table 3 highlight that the proposed XGB method achieved the highest area under the curve (AUC) value of 0.94, surpassing KNN with an AUC of 0.89. Additionally, our method demonstrated a notable 4.55% improvement over KNN for DYI, where a score of 1 indicates optimal performance.

**Figure 5:**
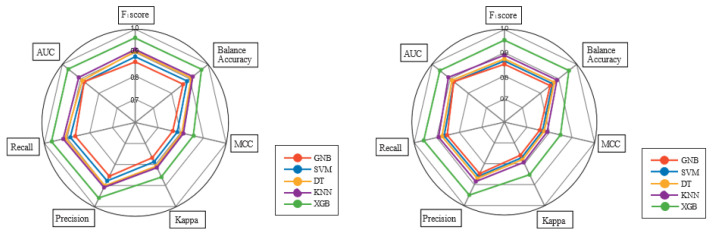
The figure shows the results of both validation (**right**) and training (**left**) phases in a radar graph.

In contrast, the radar graph provides a unique perspective on how metrics are distributed across training and test datasets. The graph represents a perfect score as a circle that completely covers the grid. Our study indicates that the training sets exhibit high scores, whereas the test set scores are relatively lower. The shape of the graphs can also serve as an indicator of model quality, with a larger circle for the test set suggesting a better-performing model. As shown in Figure 5, the proposed system generates similar pie charts for both the training and test sets, indicating a well-balanced model performance. This can be attributed to the system’s high generalizability, meaning it achieves optimal training without suffering from overfitting or underfitting. Consequently, the system provides accurate output for new inputs, while the Gaussian Naive Bayes (GNB) method demonstrates inferior performance across most metrics.

Based on the results obtained, it can be concluded that the proposed system demonstrates a high level of accuracy in automatically classifying patients with schizophrenia. The findings validate the effectiveness of the system in accurately identifying and categorizing individuals with this mental disorder, emphasizing its potential as a valuable tool for assisting in the diagnostic process of schizophrenia.

## 5. Discussion

Discriminating schizophrenia poses a non-trivial classification challenge, necessitating the use of robust and powerful optimization algorithms. Additionally, an efficient feature selection procedure is required to automate the detection process effectively. By employing these techniques, it becomes possible to investigate the underlying physiopathology of schizophrenia, which can aid in prognosis and treatment decision-making. These approaches contribute to a better understanding of the condition and enable more targeted and personalized interventions for individuals with schizophrenia [6].

Electrophysiological measurements serve as a valuable tool in aiding diagnostic decisions, particularly when cognitive symptoms are involved. Technologies such as electroencephalography (EEG), magnetoencephalography (MEG), and functional imaging are employed to investigate cognitive processes like memory, perception, and language in healthy individuals. However, their utility becomes even more significant when cognitive symptoms are part of the diagnostic criteria. EEG, in particular, is extensively utilized for analyzing cognitive functions such as visual scene perception, making it well-established for studying diseases like schizophrenia where cognitive symptoms manifest prominently [5,6,55].

Several studies have demonstrated the versatility of the XGB method in various ML applications, particularly in the medical field. Examples include its application in classifying patients with cancer [56], epilepsy [57], renal diseases [58], atrial fibrillation [59], localizing sub-mitochondrial proteins [60,61], and more. The XGB method’s favorable characteristics in terms of execution speed and scalability [37] make it particularly well-suited for this study.

Other works rely on the Super Vector Machine (SVM) algorithm. In [62], the SVM algorithm was employed for multivariate pattern analysis of microstate features. The analysis identified three patterns of correlated features, resulting in an accuracy of 82.7% for group separation. Ref. [27] extracted sensor-level and source-level features from EEG signals recorded during an auditory oddball task, achieving higher classification accuracy when combining source-level and sensor-level features compared to using only sensor-level features. The highest classification accuracy achieved in their study was 88.24%. Additionally, ref. [63] investigated the external validity of two SVM models by analyzing neuropsychological test performance and diagnostic classification. The first model achieved a trial accuracy classification rate of 84%, while the second model exhibited a higher accuracy rate of 87%. These studies demonstrate the application of the SVM algorithm in EEG analysis for classification and diagnostic purposes.

In other approaches, the utilization of Radio Frequency (RF) has been explored for the classification of multichannel EEG records. In [64], a model-free method was proposed for the binary classification of EEG records. The study achieved the best performance with a seven-dimensional feature space, attaining an average accuracy of 83.6%. Similarly, in [65], a multifractal-based approach achieved an overall cross-validation accuracy surpassing 89% in classifying individual cases.

Other notable approaches in achieving high accuracy values include Ensemble Bagged Tree (EBT) and Adapting Boosting (Adaboost). According to [27], the EBT approach achieved a correct classification rate of 93.21% for schizophrenia patients, with an overall accuracy of 89.59%. Conversely, ref. [34] applied Adaboost and obtained a classification accuracy of 91%. These studies highlight the effectiveness of EBT and Adaboost algorithms in accurately classifying individuals with schizophrenia based on EEG recordings.

On the other hand, it is also worth noting other approaches that are not based on ML. For instance, ref. [66] uses a CNN algorithm (deep learning, DL) instead of the proposed method (XGB). One key difference is the architecture: XGB is based on gradient boosting, whereas CNN is a type of neural network specifically designed for processing grid-like data such as images. The advantages of XGB include its interpretability and ease of use. XGB provides feature importance scores, allowing users to understand the impact of each input variable on the model’s predictions. It also offers flexibility in handling different types of data, such as numerical and categorical variables, and can handle missing values without imputation. Furthermore, XGB tends to be computationally efficient and can handle large datasets with high dimensionality. It has advanced regularization techniques that prevent overfitting and enhance generalization. In contrast, CNNs excel in capturing complex patterns and spatial dependencies in data, making them particularly well-suited for image and video analysis tasks. They automatically learn hierarchical representations from raw input data, eliminating the need for manual feature engineering. However, they have a much higher computational burden than ML techniques and this complicates their implementation in clinical services. In this study, very high accuracy values have been achieved, surpassing CNN techniques. Because of all this, ML techniques were selected in this study for greater flexibility in implementation, computational load, scalability, and the proposed method achieves very high accuracy values.

In this study, pattern recognition using the XGB method was performed, yielding highly accurate discrimination between healthy controls and individuals with schizophrenia, with an impressive accuracy rate of 94.25%. This outcome highlights the potential clinical utility of the proposed XGB method for EEG data analysis. Table 2 and Table 3 present a comparison between the proposed system and commonly used ML methods. Notably, the XGB method outperforms the other techniques, demonstrating superior performance and the ability to handle high-dimensional data without overfitting. Among the compared methods, SVM and GNB exhibit poorer results and performance, while KNN shows similar accuracy values compared to the proposed method.

Table 4 provides a summary of the diagnosis of schizophrenia based on EEGs using different techniques. In [67], a computationally efficient algorithm was developed, incorporating various features such as autoregressive (AR) model parameters, band power, fractal dimension, and wavelet energy. By employing Linear Discriminant Analysis (LDA) and a bidirectional search for channel selection, an accuracy of 84.62% was achieved, while using the LRS technique for channel selection resulted in an accuracy of 88.23%. These results demonstrate improved classification accuracy and relatively low computational time using the two-stage feature selection approach compared to single-stage (evolutionary feature selection) and Principal Component Analysis (PCA)-based feature selection methods.

These studies not only highlight the usefulness of EEG signals in distinguishing between individuals with schizophrenia and control participants but also indicate that the proposed method achieves higher accuracy and significantly enhances performance in this regard. Consequently, it can be concluded that the proposed system serves as a reliable and precise tool for automated EEG analysis in the diagnosis of schizophrenia. The improved accuracy and performance of the system provide promising prospects for its practical application in clinical settings for schizophrenia diagnosis.

The study has some limitations, mainly due to sample characteristics. The patients are heterogeneous in clinical aspects, duration of the illness, or duration and dosage of antipsychotic treatment, all of which could have an impact on the EEG recording. That is why the influence of different factors on EEG recording is being analyzed, as well as how to achieve better EEG quality. This will allow us to obtain improved results.

As for the future lines of the study, other types of mental health pathologies could be analyzed, specifically bipolar disorder and depression. Likewise, deep learning techniques could be applied in the study of mental health patients after the registration of magnetic resonance images.

## 6. Conclusions

In this study, four ML algorithms, namely KNN, DT, GNB, and SVM, were compared with the proposed XGB method. The performance evaluation was conducted using a 10-fold cross-validation process. The results demonstrated that the proposed XGB method outperformed the other algorithms, achieving a higher prediction accuracy of 94%, along with high precision and recall values (>0.94).

While KNN, SVM, and SVM also exhibited moderate prediction accuracy (>86), recall (>0.86), and precision (>0.86), the XGB method surpassed them in terms of prediction accuracy. These findings highlight the superior performance of XGB in terms of precision, recall, and accuracy compared to the other analyzed methods, affirming its reliability for the automatic classification of schizophrenia.

In conclusion, the XGB-based system proved to be valid and reliable, providing valuable support to clinicians in the clinical diagnosis of schizophrenia. The system’s high accuracy and robust performance make it a valuable tool in assisting with the evaluation and diagnosis of the targeted pathology discussed in this study.

## Figures and Tables

**Table 1:** The table displays the demographic data of the study participants.

	SC N = 312	SC-SUD + N = 128	SC-SUD-N = 184	HC N = 320
Gender Female	97 (31.1%)	39 (30.4%)	58 (31.5%)	144 (45.0%)
N (%)				
	**Mean (SD)**			**Mean (SD)**
Age (years)	36.6 (9.7)	35.6 (9,5)	38.4 (9.1)	36.2 (12.4)
Age of onset	23.8 (6.0)	23.2 (5.1)	24.2 (5.2)	-
(years)				
Duration of ill-	12.40 (9.3)	11.56 (9.2)	15.2 (8.6)	-
ness (years)				
Antipsychotic	Atypical: 282 (90.4%)	Atypical: 116 (90.6%)	Atypical: 167 (90.7%)	-
	Typical: 30	Typical: 12	Typical: 17	
	(9.6%)	(9.4%)	(9.3%)	
Educational	12.5 (3.9)	12.1 (3.5)	12.8 (4.1)	12.9 (3.8)
level [years]				
PANSS-T	54.3 (15.8)	54.9 (16.3)	54.2 (16.1)	-
PANSS-P	11.7 (4.3)	12.4 (6.3)	10.8 (5.1)	-
PANSS-N	16.8 (7.1)	17.3 (7.6)	16.5 (8.1)	-
PANSS-PG	19.3 (3.6)	20.2 (3.7)	19.8 (3.5)	-

SC: schizophrenia group; HC: Healthy control group; PANSS-N = Positive and Negative Syndrome Scale–Negative symptoms; PANSS-P = Positive and Negative Syndrome Scale–Positive symptoms; PANSS-PG = Positive. and Negative Syndrome Scale–General psychopathology; PANSS-T: Positive and Negative Syndrome Scale total score; SD: Standard deviation.

**Table 2:** The table resumes the results achieved by different ML models compared with XGB.

Methods	Recall	Accuracy	*F*_1_ Score	Precision
SVM	86.57 ± 0.79	86.86 ± 0.74	86.67 ± 0.76	85.69 ± 0.75
DT	87.48 ± 0.76	87.76 ± 0.82	87.23 ± 0.79	86.59 ± 0.81
GNB	85.61 ± 0.83	85.64 ± 0.74	85.25 ± 0.73	85.02 ± 0.69
KNN	89.63 ± 0.52	89.54 ± 0.61	89.07 ± 0.42	88.75 ± 0.39
XGB	94.51 ± 0.26	94.25 ± 0.28	94.92 ± 0.30	94.62 ± 0.27

**Table 3:** The table shows the results of different indicators of the ML system and the proposed model evaluated.

Methods	DYI	AUC	Kappa	MCC
SVM	86.47 ± 0.71	0.86 ± 0.02	76.54 ± 0.67	75.74 ± 0.68
DT	87.53 ± 0.68	0.87 ± 0.02	76.92 ± 0.73	77.62 ± 0.78
GNB	85.28 ± 0.74	0.85 ± 0.02	75.62 ± 0.74	74.71 ± 0.83
KNN	89.71 ± 0.58	0.89 ± 0.02	79.83 ± 0.59	79.23 ± 0.56
XGB	94.26 ± 0.23	0.94 ± 0.02	91.53 ± 0.27	91.12 ± 0.24

**Table 4:** The table summarizes the results of various published ML methods compared to the proposed system.

Author	Feature Extraction	Classifier	ACC (%)
Sabeti et al. [67]	Autoregression, band power	LDA	88.23
Johannesen et al. [63]	Rhythms separated using filtering	SVM	87.00
Baradits et al. [62]	Multivariate pattern analysis	SVM	82.07
Piryatinska et al. [64]	*e* complexity vector	RF	83.60
Siuly, et al. [68]	EMD, five statistical features	EBT	89.59
Racz, et al. [65]	Graph-based features	RF	89.29
Shim et al. [27]	Combined sensor and source level EEG features	SVM	88.24
Sabeti et al. [34]	Entropy, complexity	Adaboost	91.00
Proposed	Higuchi, Band power, DFA, Hurst	XGB	94.25

## Data Availability

The datasets used and/or analyzed during the present study are available from the corresponding author on reasonable request.
